# Estradiol replacement attenuates alendronate‐associated adverse effects on alveolar bone repair in ovariectomized rats

**DOI:** 10.1002/jper.70088

**Published:** 2026-02-19

**Authors:** Pedro H. C. Isaias, Paulo G. B. Silva, Isabelly V. do Nascimento, Raul A. D. A. da Silva, Thayla M. do C. Sousa, Ana J. A. de Vasconcelos, José V. M. Lemos, Fabrício B. Sousa, Ana P. N. N. Alves, Mário R. L. Mota

**Affiliations:** ^1^ Department of Dental Clinic Postgraduate Program in Dentistry, Federal University of Ceará (UFC) Fortaleza Ceará Brazil; ^2^ Department of Dentistry Centro Universitário Inta (UNINTA), Fortaleza Campus Fortaleza Ceará Brazil; ^3^ Department of Dentistry Universidade Christus (Unichristus) Fortaleza Ceará Brazil; ^4^ Ceará Oncology School Ceará Cancer Institute (ICC) Haroldo Juaçaba Hospital Fortaleza Ceará Brazil; ^5^ Department of Dental Clinic Faculty of Pharmacy Dentistry and Nursing, Federal University of Ceará (UFC) Fortaleza Ceará Brazil

**Keywords:** alendronate, bisphosphonate‐associated osteonecrosis of the jaw, bone remodeling, estradiol, osteoporosis, ovariectomy, tooth extraction

## Abstract

**Background:**

Sodium alendronate (ALN) is widely used to treat postmenopausal osteoporosis, but both estrogen deficiency and antiresorptive therapy may impair alveolar bone repair after tooth extraction. A clinically relevant but insufficiently explored scenario involves estrogen‐deficient individuals receiving hormone replacement therapy (HRT) who subsequently initiate ALN treatment. This study investigated whether estradiol valerate (E) replacement attenuates ALN‐associated disturbances in alveolar bone repair in ovariectomized (OVX) rats.

**Methods:**

Sixty‐three female Wistar rats were divided into sham‐operated (SHAM) or OVX groups and treated with ALN (5 or 10 mg/kg) or 0.9% sodium chloride saline solution (SAL), with or without E replacement (0.8 mg/kg). The left first molar was extracted, and alveolar repair was evaluated 28 days later through clinical, radiographic, histological, and immunohistochemical (tumor necrosis factor alpha [TNF‐α], receptor activator of nuclear factor kappa‐B ligand [RANKL], osteoprotegerin [OPG], and tartrate‐resistant acid phosphatase [TRAP]) analyses.

**Results:**

OVX increased body mass and reduced uterine wet mass (*p* < 0.001). Radiographically, ALN‐treated groups exhibited increased socket radiolucency, particularly at the higher dose (*p* = 0.040). Microscopic analysis revealed more empty osteocyte lacunae (*p* = 0.012) and connective tissue (*p* = 0.010), particularly in ALN‐treated OVX groups. OVX and ALN increased TNF‐α, RANKL, and TRAP expression and reduced OPG levels (*p* = 0.004; = 0.002; = 0.030; < 0.001). E replacement mitigated these effects and reduced type III collagen deposition (*p* < 0.001).

**Conclusions:**

Chronic ALN impaired alveolar bone repair, exacerbated by estrogen deficiency. HRT with E partially attenuated these effects without fully restoring healing. The persistent remodeling disturbances highlight the relevance of hormonal status for dental risk assessment in patients receiving bisphosphonates.

**Plain language summary:**

Osteoporosis is common in postmenopausal women due to reduced estrogen levels and leads to weaker bones. Alendronate is widely prescribed to lower fracture risk, but it may interfere with bone healing after dental procedures such as tooth extraction. Using a rat model that mimics postmenopausal bone loss, this study examined how estrogen deficiency, alendronate treatment, and estrogen replacement together influence healing of the tooth socket, a process highly relevant to periodontal and oral surgical care. The results showed that estrogen deficiency alone already compromised bone repair, and this effect became more pronounced when alendronate was used. Estrogen replacement with estradiol valerate did not fully restore normal healing, but it reduced several harmful changes in bone structure and local inflammatory activity associated with alendronate. Importantly, no bone necrosis was observed under the conditions studied, although persistent disturbances in bone remodeling were evident. These findings help clarify how systemic bone conditions and osteoporosis treatments can influence periodontal wound healing and support more informed risk assessment and interdisciplinary planning when dental extractions are required in patients with osteoporosis.

## 1 INTRODUCTION

Osteoporosis is a common systemic condition in postmenopausal women, characterized by reduced bone mass and deterioration of its microarchitecture. Estrogen deficiency worsens bone loss by increasing osteoclastic activity without a corresponding increase in osteoblastic activity, heightening fracture risk, and delaying bone repair.^1^ Bisphosphonates, such as sodium alendronate (ALN), are widely prescribed to inhibit osteoclastic bone resorption in osteoporotic patients and have proven effective in reducing fracture risk.^2^ However, concerns have emerged regarding their impact on bone repair, particularly following dental extractions, due to their association with delayed repair and an increased risk of osteonecrosis, especially in estrogen‐deficient patients.^3^, ^4^, ^5^

Beyond systemic bone loss, estrogen deficiency negatively impacts alveolar bone integrity by promoting a proinflammatory environment.^6^, ^7^, ^8^ Hormone replacement therapy (HRT) with estradiol has been employed to preserve bone mass, reduce bone resorption, and enhance bone repair by modulating the receptor activator of nuclear factor kappa‐B ligand (RANKL)/osteoprotegerin (OPG) signaling pathway, a key regulator of osteoclastic activity.^2^

A clinically relevant scenario involves postmenopausal patients who initiate HRT for systemic benefits, such as cardiovascular protection, and subsequently begin bisphosphonate therapy due to persistent fracture risk or a diagnosis of osteoporosis.^9^, ^10^ This sequential administration represents a therapeutic escalation where bisphosphonates are introduced into an environment already modulated by exogenous estrogen.^9^, ^11^ Consequently, understanding how this specific background estrogen status influences the tissue response to bisphosphonates is critical for refining risk stratification in patients undergoing oral surgery.

Although the individual effects of ALN,^5^, ^12^, ^13^, ^14^ estrogen deficiency,^6^, ^15^, ^16^, ^17^ and HRT^18^, ^19^ on bone repair are well documented, the cumulative biological impact of these concurrent therapies remain complex and unpredictable. Current literature primarily addresses these interventions in isolation or in head‐to‐head comparisons, which may not fully predict the cellular outcomes of their interaction.

Ovariectomized rats (OVX), a well‐established model of postmenopausal osteoporosis,^20^ serve as a valuable platform for investigating the effects of ALN and estradiol on bone repair and metabolism. Therefore, the aim of this study was to evaluate the effect of HRT with estradiol on alveolar bone repair following tooth extraction in rats undergoing OVX chronically treated with ALN, hypothesizing that physiological estrogen levels may counteract the excessive suppression of remodeling caused by bisphosphonates.

## 2 MATERIALS AND METHODS

### 2.1 Animals, groups, and doses

The study was approved by the Ethics Committee on Animal Use of the Federal University of Ceará (protocol no. 6914030622) and adhered to the ARRIVE guidelines for animal research reporting.^21^ A total of 63 female Wistar rats, aged 8–10 weeks and weighing 180–20 g, were included. Only clinically healthy animals deemed suitable for the experimental procedures were included. The animals were housed under controlled environmental conditions with a 12‐h light/dark cycle. Environmental enrichment included shredded paper for foraging and polyvinyl chloride (PVC) tubes for hiding. Throughout the experimental period, animals had ad libitum access to food and water, allowing ovariectomy‐associated weight gain that reflects a common postmenopausal characteristic.^22^ Body weight was recorded weekly. The number of animals was determined based on the study by Watanabe et al. (2020)^15^ which reported significant differences in bone mineral density between OVX and control groups. Using their data, a sample size of 7 animals per group was calculated to achieve 90% power and 95% confidence, with a 5% significance level.

**FIGURE 1:**
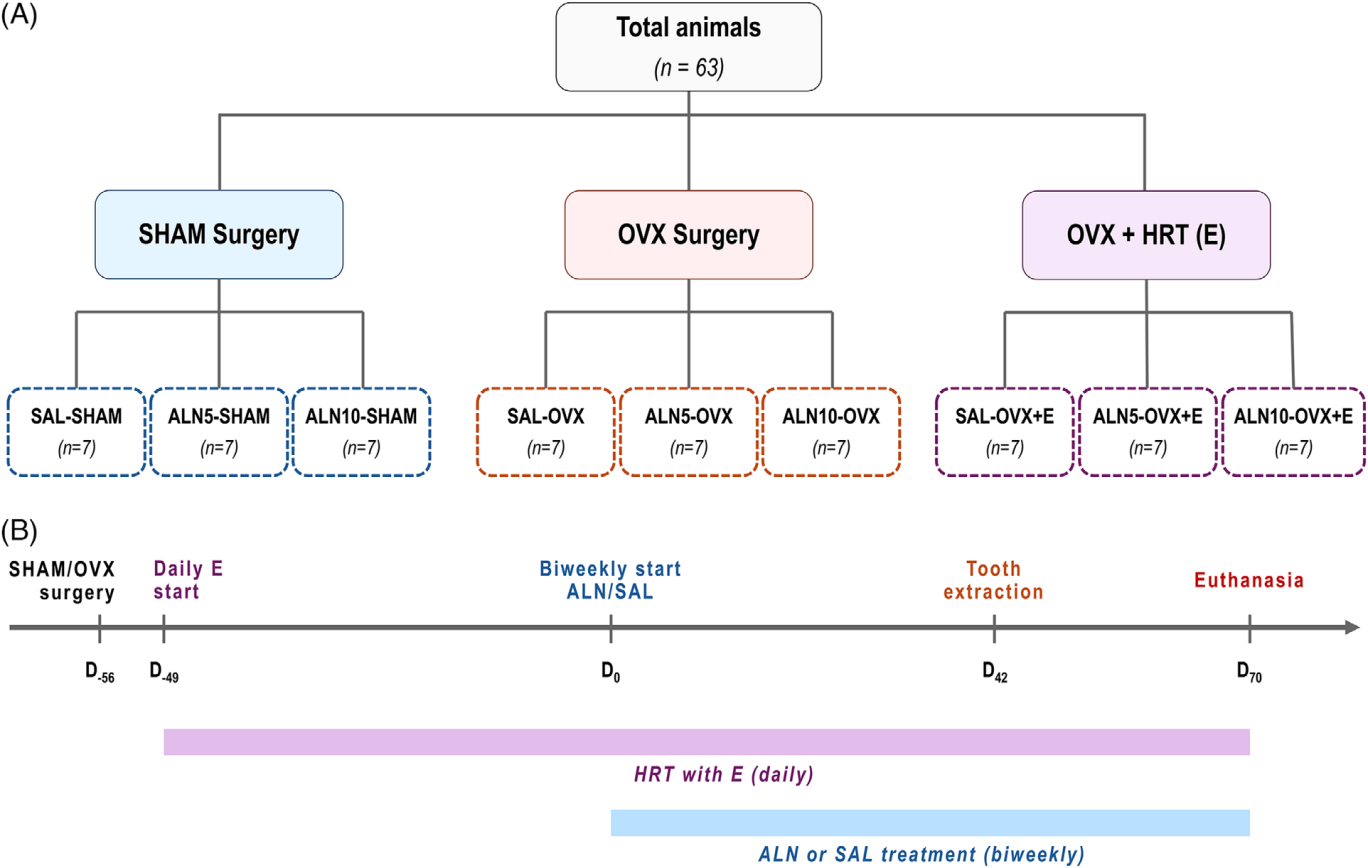
Experimental design timeline and animal group distribution. The flowchart (A) details the allocation of 63 rats into the 9 final experimental groups (*n* = 7 each) based on the 3 × 3 factorial design (surgical/hormonal status × drug treatment). The timeline (B) illustrates the sequence of procedures: OVX or SHAM surgery was performed on day ‐56, followed by the initiation of HRT with E or vehicle administration on day ‐49. ALN (5.0 or 10.0 mg/kg) or SAL (1.0 mg/kg) administration commenced on day 0. Tooth extraction was performed on day 42. Euthanasia and sample collection occurred on day 70, representing the 28‐day postextraction endpoint. ALN5, sodium alendronate 5 mg/kg; ALN10, sodium alendronate 10 mg/kg; E, estradiol valerate 0.8 mg/kg; HRT, hormonal replacement treatment; OVX, ovariectomy; SAL, saline (vehicle control 1.0 mg/kg); SHAM, sham‐ovariectomy

The rats were randomly allocated into 9 experimental groups using a random number generator before treatment began to minimize bias. OVX groups underwent bilateral removal of the ovaries to simulate estrogen deficiency, while sham‐operated (SHAM) groups underwent a simulated surgical procedure, with ovaries exposed and replaced in the abdominal cavities, on day D_‐56_.^23^ Estradiol valerate (E) replacement was initiated 1‐week after OVX (D_‐49_) to mimic the early onset of HRT following menopause. After an 8‐week recovery period, allowing the rats to develop osteoporotic characteristics due to estrogen deficiency,^20^ the rats in the treatment groups received ALN or saline (SAL) starting on D_0_, simulating the introduction of bisphosphonate therapy in patients already undergoing HRT, to assess their individual and combined effects on alveolar bone repair. ALN (Delta) was administered at 5 and 10 mg/kg, doses calculated via body surface area conversion from the standard human regimen (70 mg/week).^24^, ^25^ This protocol was chosen to simulate chronic exposure and suppress bone turnover without inducing overt osteonecrosis, consistent with established rodent models.^4^, ^5^, ^26^ HRT with E (Bayer) was administered by gavage at a dosage of 0.8 mg/kg/day, starting 1 week after OVX surgery.^27^ The 9 total groups are described in Figure 1A

### 2.2 Experimental protocol

Following a tooth extraction protocol for ALN‐treated rats,^5^ SAL control groups received 0.9% sodium chloride solution by gavage, twice weekly over 10 weeks (D_0_‐D_63_). ALN groups received bisphosphonate under the same schedule. Tooth extractions were performed on day 42 to allow for a sufficient period of drug exposure and accumulation, in a sterile environment, specifically targeting the lower left first molar under anesthesia (ketamine 80 mg/kg and xylazine 10 mg/kg, intraperitoneally). Both procedure duration and root fracture incidence were recorded in a blinded manner. Animals were monitored daily for signs of pain, distress, or discomfort, such as weight loss, abnormal posture, or reduced appetite. The experimental protocol concluded on D_70_, 28 days post‐extraction, a time point selected to evaluate final bone consolidation,^28^ with euthanasia via anesthetic overdose (Figure 1B). Blood samples were collected to assess total leukocyte counts using a Turk solution and Neubauer chamber.^5^, ^12^ The leukocyte count analysis (Table S1) aimed to assess systemic health and rule out systemic inflammatory conditions that could confound local alveolar repair. Following euthanasia, hemimandibles were inspected for exposed bone, surgically removed, and stored in 10% buffered formalin for radiographic and microscopic analysis. Femurs were also collected for histological analysis, and uteri were removed and weighed, with values expressed as wet mass on euthanasia day.^19^ The evaluators were blinded to group allocations during clinical, radiographic, macroscopic, and microscopic analyses, as well as during statistical analysis, to prevent bias.

### 2.3 Radiographic analysis

All hemimandibles were radiographed using a conventional x‐ray device (DabiAtlante; 63 Kvp, 8 mA) coupled with a digital image capture system, employing parallelism technique. Hemimandibles were positioned parallel to the radiographic sensor, adjacent to a 5 mm metallic matrix, with a focus‐film distance of 30 cm. Exposure time was set at 0.3 s. Radiographs were converted to JPEG format for quantitative analysis using ImageJ software (National Institutes of Health, USA). A blinded operator measured the radiolucent area in triplicate using the freehand selection tool. The arithmetic mean of the 3 measurements was considered the sample unit.^5^, ^29^

### 2.4 Histological and histomorphometric analysis

Following radiographs, left hemimandibles, and femurs were decalcified using a 10% ethylenediaminetetraacetic acid (EDTA) solution (pH 7.3) for 90 and 60 days, respectively, to allow for histological processing. After decalcification, 3 µm sections were stained with hematoxylin and eosin and analyzed using light microscopy. Histological evaluation focused on the inflammatory infiltrates, microbial colonies, and histological signs of bone necrosis in the mandibles. Additionally, femoral epiphysis and periosteum were examined for pathological changes.^5^, ^29^, ^30^

Histological slides of mandibles were photographed in 10 high‐magnification fields (400×) at the surface, mid‐region, and deepest part of the first left mandibular molar extraction site, using a microscope equipped with a digital camera. These images were analyzed using ImageJ to quantify the number of empty and viable osteocyte lacunae, vital and apoptotic osteoclasts, and mononuclear and polymorphonuclear inflammatory cells. The sum of counts from all fields was considered the sample unit.^5^, ^29^ Lower magnification images (25×) were captured to measure the unfilled area with the bone tissue of the extraction site, expressed in µm^2^, from the crest of the neighboring second molar to the most apical part of the repair socket.^5^ The percentage area of bone marrow spaces in both mandible and femur was analyzed using lower magnification images (25×), binarized in ImageJ. Bone area (black pixels) was measured and subtracted from the total area of the extracted tooth socket or femur head to determine trabecular spaces, expressed as a percentage (see Figure S1) All measurements were conducted blindly, in triplicate, with the arithmetic mean serving as the sample unit.^29^

### 2.5 Histochemical analysis of collagen fibers

Collagen deposition and typification at extraction sites were assessed using Picrosirius Red staining (ScyTek) under polarized light on 3 µm sections. Five fields (200× magnification) from the region of interest were photographed using the same microscope as the histomorphometric analysis, with additional polarized light. Photomicrographs were analyzed in ImageJ. For total collagen, the average percentage of red‐stained collagen area was measured in pixels. Thick, strongly birefringent fibers (orange to red) under polarized light were identified as type I collagen, while thin, weakly birefringent fibers (green) represented type III collagen. The average percentage of each collagen type (total, type I, and type III) from the 5 fields was used as the sample unit.^29^, ^31^

### 2.6 Immunohistochemical analysis

A representative region of the surgical site was selected for new paraffin blocks using the tissue microarray (TMA) technique with the Quick‐Ray UNITMA device. A 2 mm tissue fragment was extracted from the region of interest in each donor block and transferred to the recipient block. Silanized slides (4 µm) were incubated overnight with primary antibodies: tumor necrosis factor‐alpha (TNF‐α; 1:100; ab199013, Abcam), tartrate‐resistant acid phosphatase (TRAP; 1:100; ab212723, Abcam), RANKL (1:200; ab45039, Abcam), and OPG (1:200; ab73400, Abcam). The secondary antibody (Envision System Plus‐HRP, Dako) was applied, and positive cells were visualized using 3,3′‐diaminobenzidine (ab64238, Abcam) and counterstained with Harris hematoxylin.^5^, ^29^

Five fields from each extraction site of the first mandibular molar were photographed at 400× magnification. ImageJ software was used to count cells exhibiting cytoplasmic or nuclear positivity for TNF‐α, RANKL, and OPG. TRAP‐positive osteoclasts were defined as multinucleated cells (at least 2 nuclei), with large cell bodies in direct bone contact without proximity to inflammatory tissue.^32^ The total number of positive cells in all fields was used as the sample unit for each group.

### 2.7 Statistical analysis

No data were excluded during the analysis. Quantitative variables were subjected to the Shapiro–Wilk test for normality. Variables with normal distribution were presented as mean ± standard error of the mean. Statistical analyses were performed using 1‐way or multifactor analysis of variance (ANOVA), followed by Bonferroni post‐test for group comparisons. All analyses were conducted in GraphPad Prism 5.0 software, with significance set at *p* < 0.05 for all evaluations.

The p‐value for Interaction (I) assessed whether the combined effect of OVX and ALN differed significantly from the sum of their individual effects. The OVX p‐value determined if OVX significantly impacted outcomes across groups, while the ALN p‐value indicated if ALN administration had a statistically significant effect compared to nontreated groups.

## 3 RESULTS

### 3.1 OVX and E treatment effects on body, uterine mass, and femur morphology

All animals met the inclusion criteria and completed the experimental protocol, resulting in a final sample size of 63 rats. OVX increased body mass from day 28 to day 70 in all OVX groups, regardless of ALN or E treatment, but E did not significantly reduce this weight gain (OVX *p* < 0.001). OVX also significantly reduced uterine wet mass, with E treatment mitigating this reduction, though levels remained below those of controls (OVX *p*‐value < 0.001). Histologically, OVX animals exhibited fibrous tissue with sparse osteoclast presence in the femoral epiphysis and increased bone marrow area in the femoral head. ALN treatment at both 5 and 10 mg/kg doses partially reversed this effect but did not restore normal bone structure (I *p*‐value = 0.001; OVX *p*‐value < 0.001; ALN *p*‐value < 0.001) (see Figures S2, S3 and Table S1)

### 3.2 Impact of ALN on radiographic, clinical, hematological, and surgical outcomes

**FIGURE 2:**
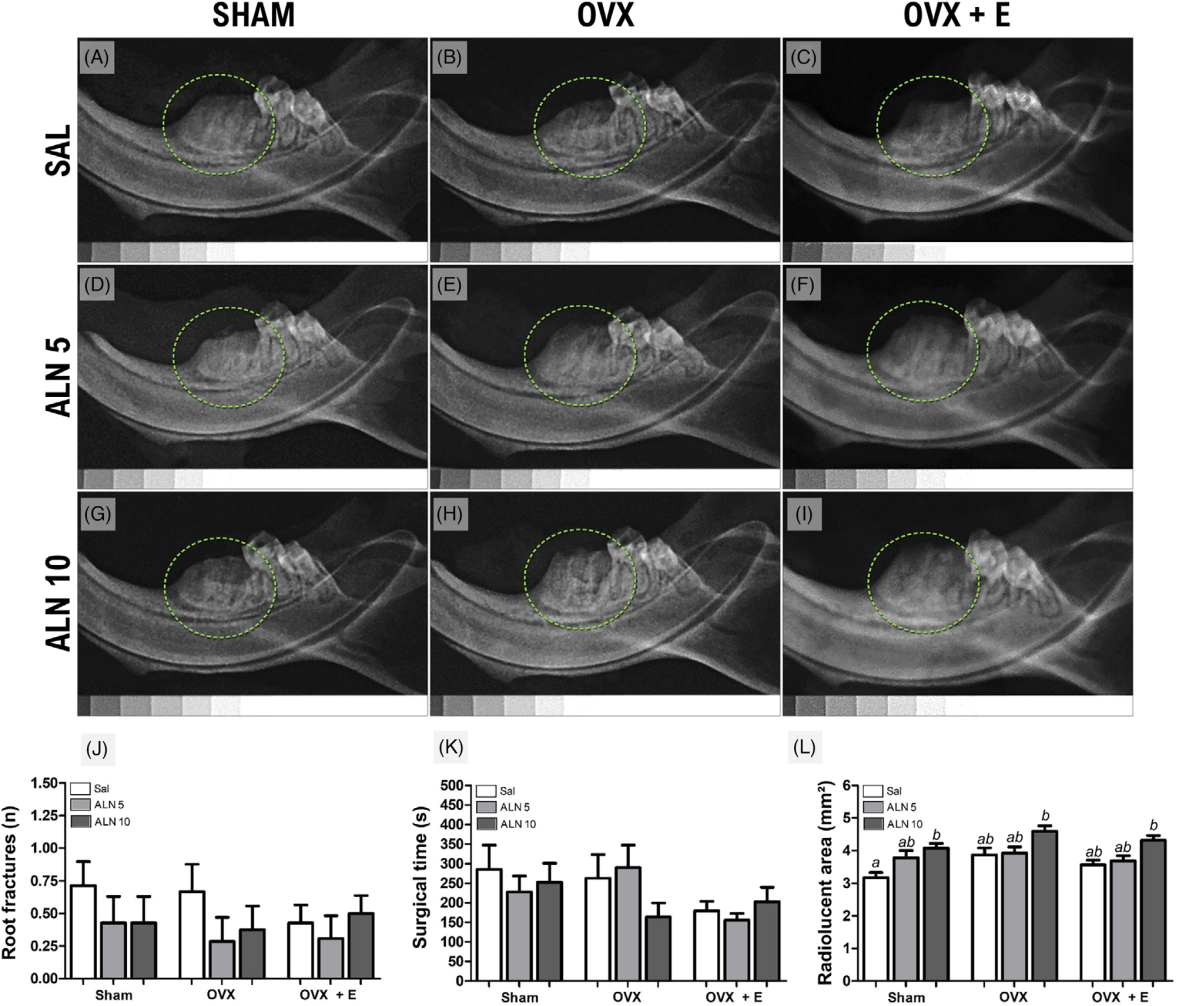
Radiographic and macroscopic assessment of experimental groups. Radiographic images (A–I) depict representative regions of the dental alveoli (dashed circles) in the left hemimandibles of OVX and SHAM rats subjected to tooth extraction, with or without treatment. Treatments included SAL or ALN (5.0 or 10.0 mg/kg), administered with or without HRT using E. The graphs present quantitative analysis of root fractures (J), surgical time (K), and radiolucent bone area (L). Data were analyzed using 1‐way and 2‐way ANOVA followed by Bonferroni post‐test. Different lower‐case letters indicate statistically significant differences between groups (*p* < 0.05). Groups that share at least 1 letter are not significantly different from each other. ALN, sodium alendronate; ANOVA, analysis of variance; E, estradiol valerate; HRT, hormonal replacement treatment; OVX, ovariectomy; SAL, saline (vehicle control 1.0 mg/kg); SHAM, sham‐ovariectomy

Clinically, all animals displayed proper extraction site repair with no bone exposure or visible complications. Extraction duration and root fracture incidence were similar across groups, and total leukocyte counts showed no significant intergroup differences (Figure 2J,K and Table S1) Radiographically, ALN‐treated and OVX groups exhibited increased radiolucency around the left mandibular molar sockets (Figure 2 A–I). Specifically, higher ALN doses significantly elevated the radiolucent area in the ALN10 groups (ALN10‐SHAM, ALN10‐OVX, and ALN10‐OVX+E) compared to SAL‐SHAM (ALN p‐value = 0.040; see Figure 2L).

### 3.3 ALN delays bone repair post‐extraction, exacerbated by OVX but mitigated by E

**FIGURE 3:**
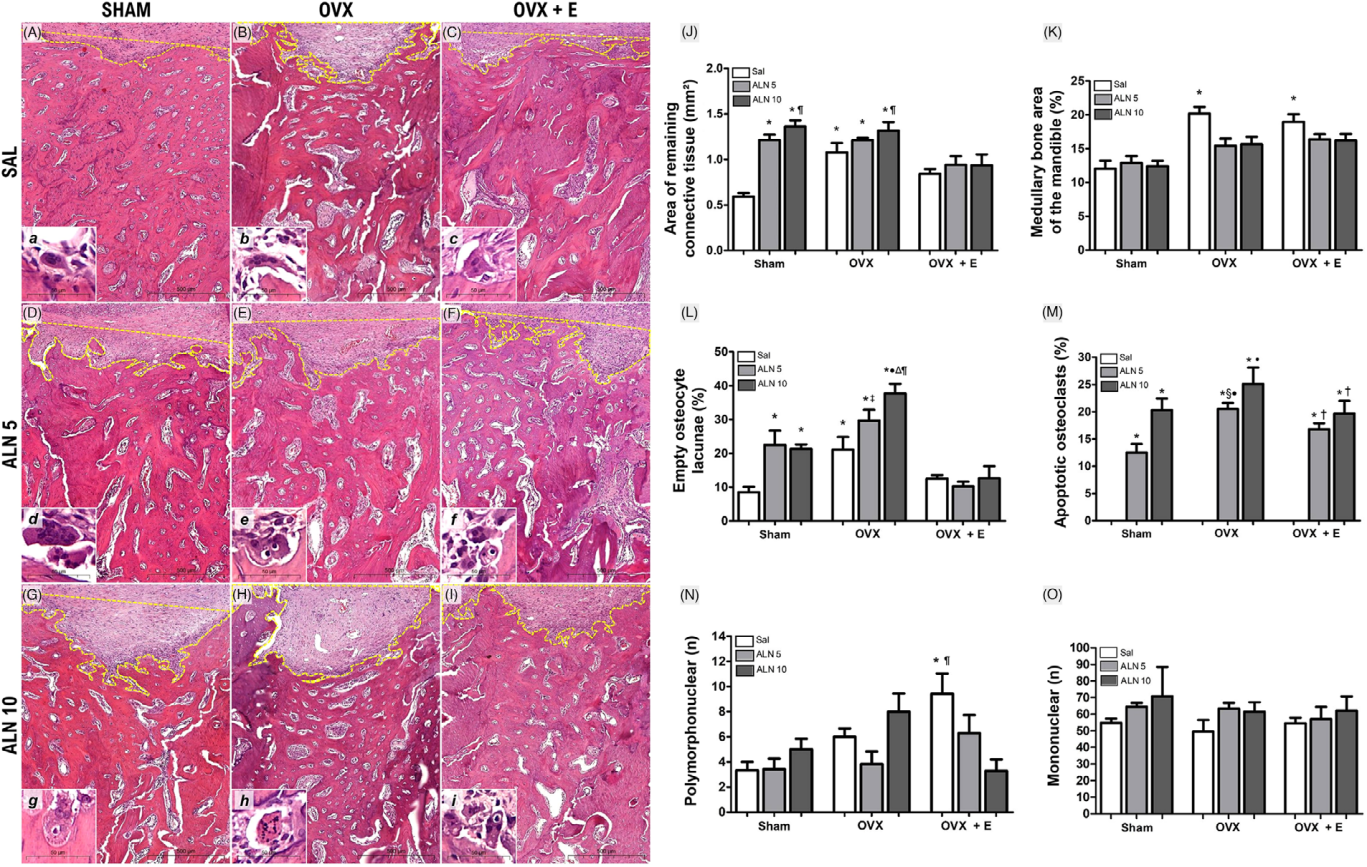
Histological analysis of tooth extraction sites. Representative photomicrographs of the dental alveoli (A–I, hematoxylin and eosin, 100×) in the left hemimandibles of OVX and SHAM rats treated with SAL, ALN (5.0 or 10.0 mg/kg), and/or E. The images highlight the fibrous connective tissue filling the alveoli (dashed lines). Magnified views (A–I, hematoxylin and eosin, 400×) show osteoclasts. The graphs (J–O) show the histomorphometric evaluation of the following parameters: area of remaining connective tissue (J), medullary bone area (K), empty osteocyte lacunae (L), apoptotic osteoclasts (M), polymorphonuclear cells (N), and mononuclear cells (O). ^*^ *p* < 0.05 vs. SAL‐SHAM group; ^●^ *p* < 0.05 vs. SAL‐OVX group; ^†^ *p* < 0.05 vs. SAL‐OVX+E group; ^§^ *p* < 0.05 vs. ALN5‐SHAM group; ^Δ^ *p* < 0.05 vs. ALN10‐SHAM group; ^‡^ *p* < 0.05 vs. ALN5‐OVX+E group; ^¶^ *p* < 0.05 vs. ALN10‐OVX+E group, 2‐way ANOVA with Bonferroni post‐test. ALN, sodium alendronate; ANOVA, analysis of variance; E, estradiol valerate; OVX, ovariectomy; SAL, saline (vehicle control 1.0 mg/kg); SHAM, sham‐ovariectomy

Microscopic analysis at 28 days postextraction revealed distinct histological patterns among the groups. In the SAL‐SHAM and OVX+E groups (Figure 3A,C), alveolar filling with viable bone and minimal inflammation was observed. In contrast, animals treated with ALN (5 and 10 mg/kg) exhibited persistence of fibrous connective tissue (Figure 3D,E,G,H). ALN treatment significantly increased the area of remaining connective tissue within the socket. This effect was exacerbated by the OVX condition, being most pronounced in the ALN10‐SHAM and ALN10‐OVX groups. HRT with E significantly mitigated this ALN‐ induced increase, reducing the fibrous tissue area in the treated groups (I *p*‐value = 0.001; OVX *p*‐value < 0.001; ALN *p*‐value = 0.0002; Figure 3J). Regarding bone marrow, OVX was associated with increased marrow spaces (OVX *p*‐value = 0.013), whereas ALN treatment significantly reduced the marrow area in the OVX and OVX+E groups (ALN *p*‐value < 0.001; Figure 3K).

ALN treatment resulted in alterations in cell viability, evidenced by a significant increase in empty osteocyte lacunae (I *p*‐value = 0.012; OVX *p*‐value = 0.001; ALN *p*‐value < 0.001; Figure 3L) and the presence of osteoclasts with intracellular vacuolization, particularly in OVX groups (OVX *p*‐value < 0.001; ALN *p*‐value = 0.0063; Figure 3 M). E replacement partially attenuated these effects: there was a significant reduction in the number of empty lacunae and vacuolated/apoptotic osteoclast counts in E‐treated ALN groups compared to their counterparts without HRT (I *p*‐value = 0.012; OVX *p*‐value = 0.001; ALN *p*‐value < 0.001).

The inflammatory infiltrate was predominantly mononuclear across all groups, with no statistically significant differences among them (Figure 3O). Regarding polymorphonuclear cells, SAL‐OVX+E group exhibited higher counts compared to SAL‐SHAM, while ALN10‐OVX+E reduced the cell counts relative to SAL‐OVX+E (I *p*‐value = 0.003; Figure 3N).

**FIGURE 4:**
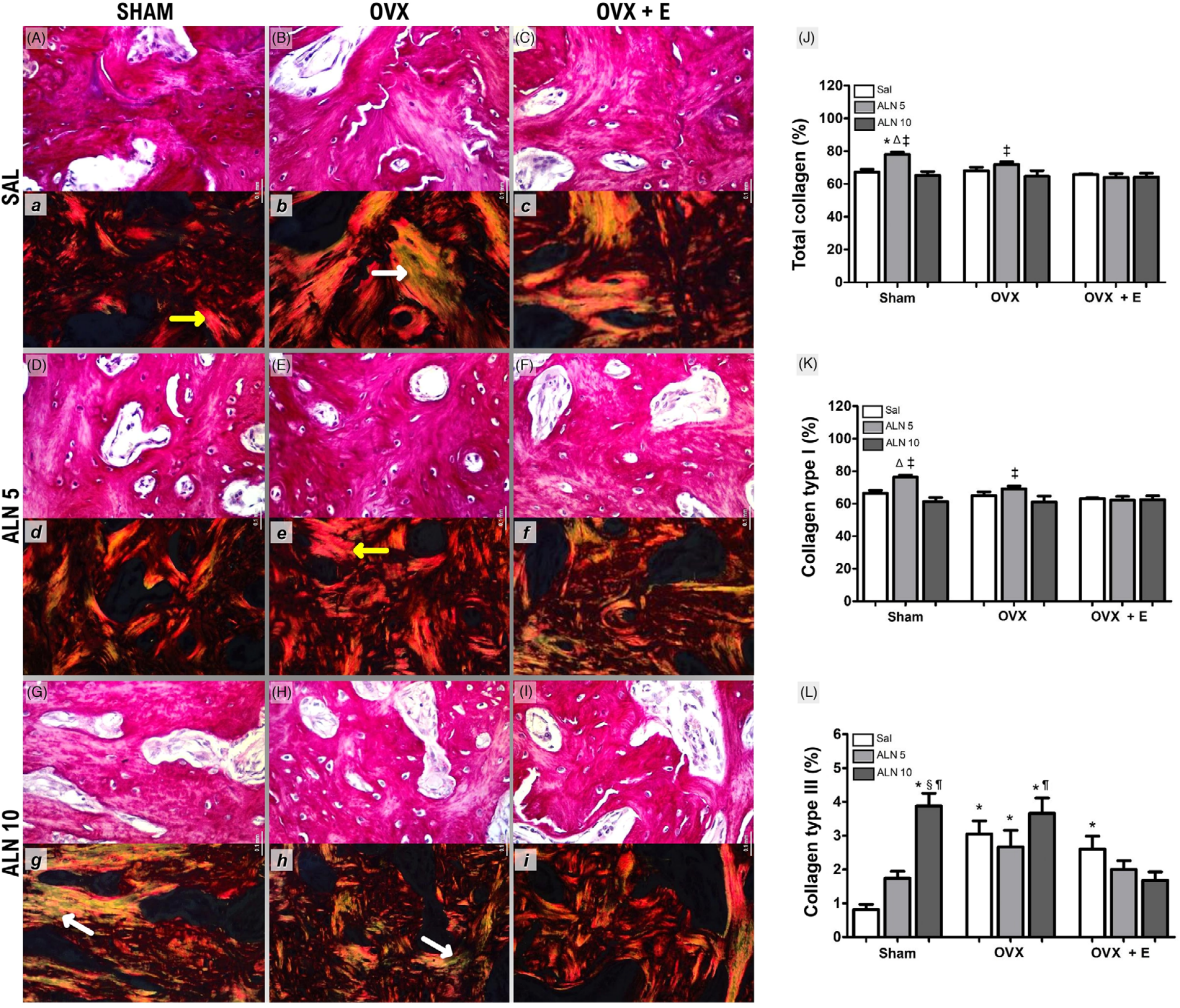
Histological analysis of collagen fibers. Representative photomicrographs of dental alveoli stained with Picrosirius Red show the effects of different treatments in OVX and SHAM rats. Images A–I display total collagen under non‐polarized light (200×). Images A–I show collagen fibers under polarized light (200×), with yellow arrows indicating type I collagen and white arrows indicating type III collagen. The graphs present the histomorphometric evaluation of total collagen (J), type I collagen (K), and type III collagen (L). ^*^ *p* < 0.05 vs. SAL‐SHAM group; ^§^ *p* < 0.05 vs. ALN5‐SHAM group; ^Δ^ *p* < 0.05 vs. ALN10‐SHAM group; ^‡^ *p* < 0.05 vs. ALN5‐OVX+E group; ^¶^ *p* < 0.05 vs. ALN10‐OVX+E group, 2‐way ANOVA with Bonferroni post‐test. ALN, sodium alendronate; ANOVA, analysis of variance; E, estradiol valerate; OVX, ovariectomy; SAL, saline; SHAM, sham‐ovariectomy

ALN treatment significantly increased total collagen deposition in ALN5‐SHAM relative to SAL‐SHAM, ALN10‐SHAM, and ALN5‐OVX+E (I *p*‐value = 0.022; OVX *p*‐value < 0.001; ALN *p*‐value = 0.011). Similarly, type I collagen was elevated in ALN5‐SHAM compared to ALN10‐SHAM and ALN5‐OVX+E (I *p*‐value = 0.014; OVX *p*‐value < 0.001; ALN *p*‐value = 0.015). Type III collagen was higher in SAL‐OVX and SAL‐OVX+E than in SAL‐SHAM, with ALN further increasing it at higher doses. E treatment partially reduced type III collagen levels in ALN‐treated OVX animals (I *p*‐value < 0.001; OVX *p*‐value = 0.002; ALN *p*‐value < 0.001; see Figure 4).

### 3.4 ALN treatment in OVX‐groups increased TNF‐α, RANKL, and TRAP levels, with reduced OPG, while E attenuated these effects

**FIGURE 5:**
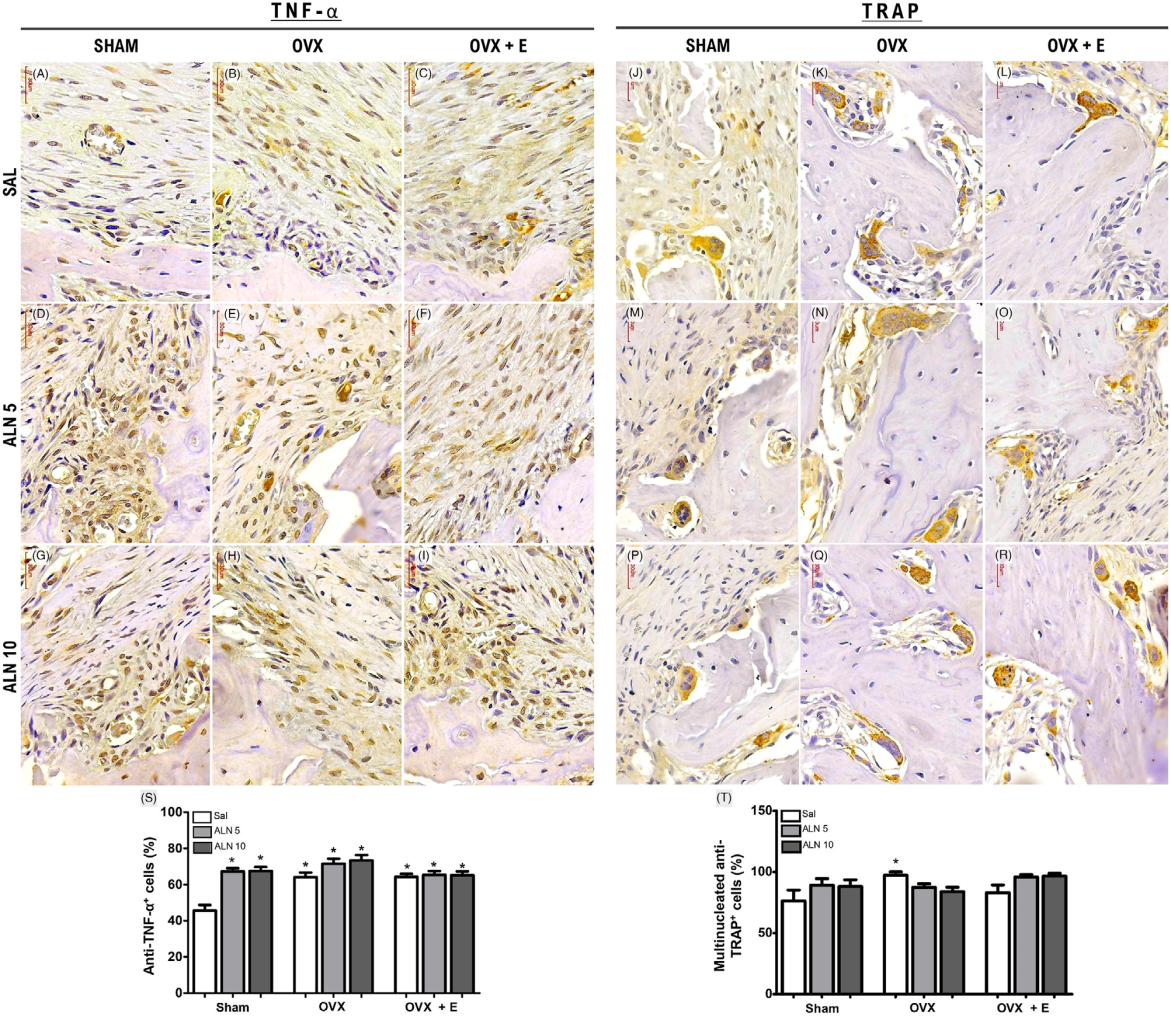
Immunohistochemical analysis for TNF‐α and TRAP. Representative photomicrographs of dental alveoli stained with antibodies against TNF‐α (A–I, 400×) and TRAP (J–R, 400×), in OVX and SHAM rats subjected to tooth extraction and treated with SAL, ALN (5.0 or 10.0 mg/kg), and/or E. TNF‐α positivity was detected in perialveolar connective tissue cells in the OVX and SHAM groups treated with ALN at both doses, while TRAP staining was observed in the membranes and cytoplasm of osteoclasts, with notably higher levels in the OVX‐treated groups. The graphs show the quantitative analysis of anti‐TNF‐α positive cells (S) and multinucleated anti‐TRAP‐positive cells (T). Statistical analysis revealed significant effects for interaction (I *p* = 0.004), OVX status (*p* = 0.001), and ALN treatment (*p* < 0.001) for TNF‐α; and a significant interaction (I *p* = 0.030) for TRAP. ^*^ *p* < 0.05 vs. SAL‐SHAM control group; 2‐way ANOVA with Bonferroni post‐test. ALN, sodium alendronate; E, estradiol valerate; OVX, ovariectomy; SAL, saline; SHAM, sham‐ovariectomy; TNF‐α, tumor necrosis factor‐alpha; TRAP, tartrate‐resistant acid phosphatase

The immunohistochemical analysis revealed significant interactions between hormonal status and treatment for TNF‐α (I *p*‐value = 0.004; OVX *p*‐value = 0.001; ALN *p*‐value < 0.001; Figure 5A–I,S) and TRAP (I *p*‐value = 0.030; Figure 5J–R,T). TNF‐α expression in perialveolar connective tissue cells increased with ALN and OVX; however, this effect was partially mitigated by E. Regarding TRAP‐positive osteoclasts, HRT reversed the OVX‐induced increase in the SAL‐OVX+E group, restoring values to control levels.

**FIGURE 6:**
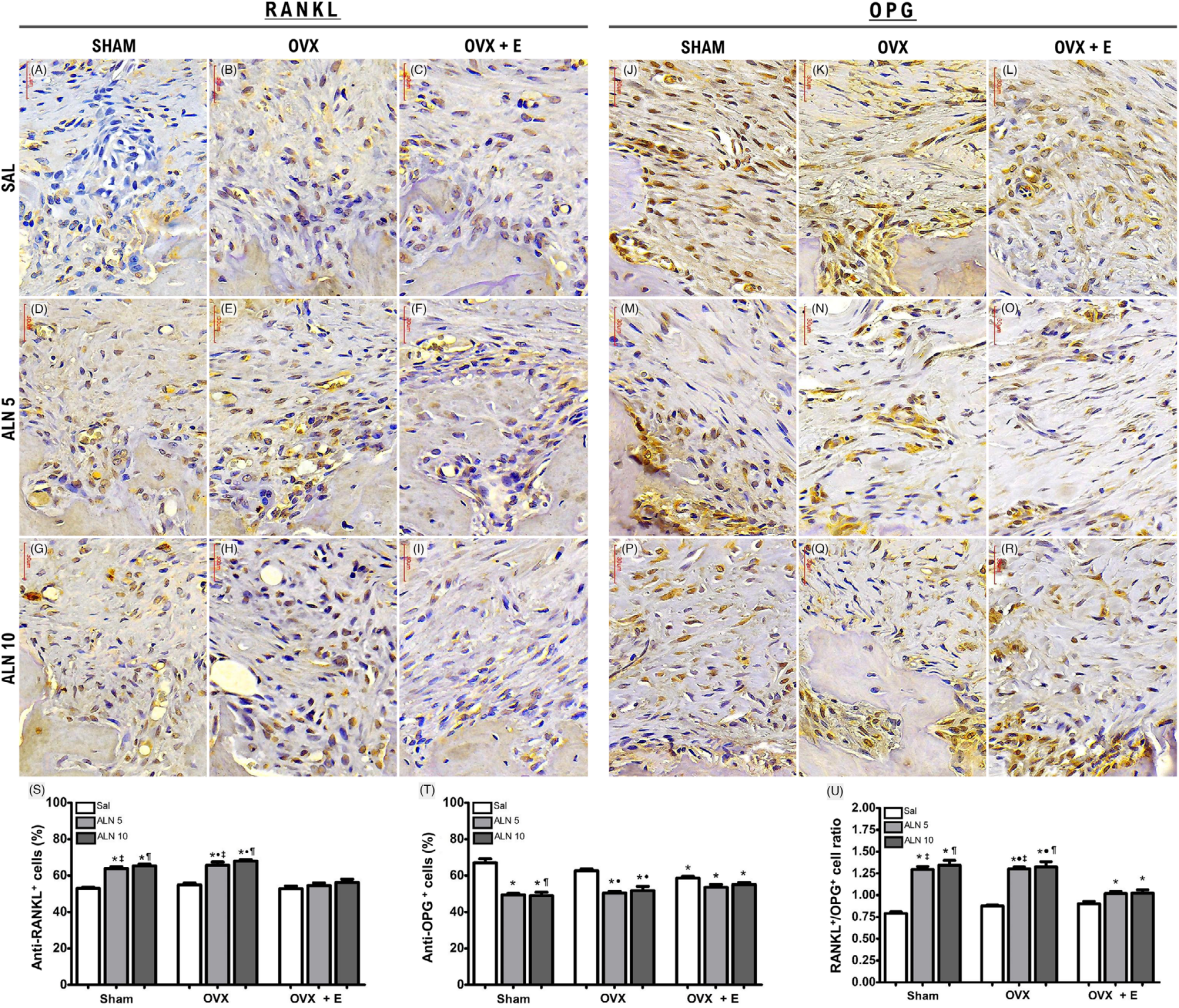
Immunohistochemical analysis of RANKL and OPG. Representative photomicrographs of dental alveoli stained with antibodies against RANKL (A‐I, 400×) and OPG (J‐R, 400×) in OVX and SHAM rats subjected to tooth extraction and treated with SAL, ALN (5.0 or 10.0 mg/kg), and/or E. RANKL immunopositivity is observed in perialveolar connective tissue cells, particularly in the ALN5‐SHAM, ALN10‐SHAM, ALN5‐OVX, and ALN10‐OVX groups. OPG immunolabeling, in contrast, is reduced in the ALN‐SHAM, ALN‐OVX, and ALN‐OVX+E groups. The graphs present the quantitative analysis of anti‐RANKL‐positive cells (S), anti‐OPG‐positive cells (T), and the RANKL/OPG ratio (U). ^*^ *p* < 0.05 vs. SAL‐SHAM group; ^●^ *p* < 0.05 vs. SAL‐OVX group; ^‡^ *p* < 0.05 vs. ALN5‐OVX+E group; ^¶^ *p* < 0.05 vs. ALN10‐OVX+E group, 2‐way ANOVA with Bonferroni post‐test. ALN, sodium alendronate; ANOVA, analysis of variance; E, estradiol valerate; OPG, osteoprotegerin; OVX, ovariectomy; RANKL, receptor activator of nuclear factor kappa‐B ligand; SAL, saline; SHAM, sham‐ovariectomy

ALN treatment raised RANKL expression in SHAM and OVX groups, with E cotreatment reducing RANKL levels in OVX+E groups (I *p*‐value = 0.002; OVX *p*‐value < 0.001; ALN *p*‐value < 0.001; see Figure 6A–I,S). OPG levels were reduced in ALN‐treated groups, but E treatment increased OPG in ALN10‐OVX+E relative to ALN10‐SHAM (I *p*‐value < 0.001; ALN *p*‐value < 0.001). The RANKL/OPG ratio was highest in ALN‐treated SHAM and OVX groups, remaining elevated even with E treatment (I *p*‐value < 0.001; OVX *p*‐value < 0.001; ALN *p*‐value < 0.001; see Figure 6J–R,T,U).

## 4 DISCUSSION

This study was the first to assess the interaction among estrogen deficiency, HRT with E, and ALN treatment — using doses allometrically scaled to human therapeutic regimens ^5^, ^24^, ^26^ — on alveolar bone repair in rats. The findings should be interpreted within this specific temporal and dosing context. On day 28, ALN was associated with reduced bone fill, especially in OVX‐induced osteopenic rats, while E replacement partially offsets these effects.

The OVX model induces osteopenia to simulate osteoporosis‐related bone loss. ALN, commonly prescribed for osteoporosis, can trigger adverse effects, such as medication‐related osteonecrosis of the jaw (MRONJ).^33^ OVX animal showed significant weight gain not reduced by E and uterine atrophy partially attenuated by E. Although OVX and low estrogen are known to elevate inflammation,^22^ there were no leukocyte count differences among treatment groups. These findings are likely due to the limited bioavailability and shorter duration of oral E and ALN treatments.^2^, ^19^, ^34^

ALN therapy is associated with surgical complications,^13^, ^35^ yet no significant extraction challenges were noted across groups, indicating that bone effects of ALN did not hinder the procedure, in concordance with other studies.^5^, ^14^ However, high‐dose ALN was associated with increased radiolucency. This pattern, alongside reduced TRAP, reflects pharmacological bone turnover suppression consistent with literature.^5^, ^6^, ^7^ Osteonecrosis‐specific lesions were absent, though ALN‐SHAM, SAL‐OVX, and ALN‐OVX groups showed increased empty osteocyte lacunae, mirroring findings from earlier studies.^5^, ^36^ Higher osteoclast apoptosis in ALN‐treated rats, particularly with OVX, suggests a compensatory response to resorption inhibition by ALN, as previously described.^5^, ^36^ E reduced these alterations, but its effect was more significant in empty lacunae than osteoclasts, suggesting its limited protective role. Estrogen deficiency is known to increase osteocyte apoptosis in both humans and rats, a process that can be reversed through HRT.^37^

Both OVX and ALN treatment (5.0 and 10.0 mg/kg) significantly raised connective tissue in dental alveoli, with high levels observed in ALN‐SHAM, ALN‐OVX, and SAL‐OVX groups, similar to findings in previous studies.^4^, ^26^ E administration reduced this effect but did not restore bone marrow spaces in the alveoli and in the femoral condyle, indicating its limitations against bone density loss. Interestingly, this protective effect was not clearly detected by radiographic analysis, suggesting that at 28 days, the mitigation provided by estradiol is more apparent at the histological level than in the overall mineral density detectable by conventional radiography, a known limitation of this technique.^5^, ^38^ No significant differences were observed in mononuclear cells, but E treatment in SAL‐OVX+E animals increased polymorphonuclear levels. Since polymorphonuclear cells typify the acute healing phase, their persistence on day 28 suggests delayed inflammatory resolution.^29^ ALN attenuated this increase, consistent with its capacity to inhibit monocyte adhesion,^39^ representing an inflammatory shift secondary to remodeling suppression rather than a proven functional benefit.

ALN and OVX raised TNF‐α, while HRT with E reduced these levels, though not significantly. TNF‐α, an inflammatory cytokine, promotes osteoclast recruitment, inhibits osteoblasts, and can induce apoptosis in these cells. The presence of empty osteocyte lacunae, indicative of osteocyte apoptosis, is associated with inflammation and inadequate bone remodeling.^40^ In OVX and ALN groups, these empty lacunae correlated with TNF‐α levels, reflecting increased bone resorption and inflammation‐associated bone loss. Moreover, the progressive rise in empty lacunae with higher doses of ALN aligns with the observed elevation in TNF‐α levels.

As expected, TRAP levels, a bone resorption marker expressed by osteoclasts,^41^ were elevated in SAL‐OVX animals, indicating increased osteoclast activity due to estrogen deficiency. This increase was attenuated by estrogen administration in SAL‐OVX+E group, supporting the role of estrogen in regulating osteoclastic activity.^16^, ^37^ ALN also reduced TRAP‐positive osteoclasts in OVX animals, reinforcing its inhibitory role in bone resorption.^42^ However, the increase in apoptotic osteoclasts observed in ALN‐treated groups suggests cellular dysfunction. Despite HRT, the suppressive effects of ALN, particularly at high doses, persisted.

Regarding the signaling pathway, ALN increased RANKL and lowered OPG, resulting in an elevated RANKL/OPG ratio independent of OVX, consistent with previous findings.^8^, ^18^ Although RANKL binding to its receptor RANK normally activates osteoclast differentiation and OPG acts as a decoy receptor,^43^ the elevated ratio observed here contrasts with the low TRAP levels. This specific molecular pattern, characterized by a high RANKL/OPG ratio (pro‐osteoclastogenic signal) alongside low TRAP (suppressed activity), is compatible with antiresorptive exposure: the signal is present, but the execution is suppressed.^7^, ^42^ This reflects a decoupled remodeling state on day 28. Importantly, this uncoupled condition, where a pro‐osteoclastogenic signal persists without corresponding resorption, can impair osteoblast differentiation and function, likely contributing to the reduced bone fill observed at this endpoint.^13^, ^43^ HRT attenuated the RANKL imbalance, bringing levels closer to the SAL‐SHAM group, reinforcing earlier findings,^18^ but did not fully restore bone homeostasis under ALN treatment.

Collagen is essential for tissue structure, regulating cell activity, initially dominated by type III, then replaced by type I.^29^ Low‐dose ALN raised total and type I collagen in SHAM animals, yet this effect was absent with higher doses, consistent with findings suggesting that moderate ALN doses can enhance collagen synthesis.^44^ Type III collagen was more pronounced in higher ALN doses, in SAL‐OVX and SAL‐OVX+E groups, correlating with TNF‐α levels and indicating impaired bone remodeling.^44^ E replacement reduced type III collagen, partly restoring normal collagen synthesis. This suggests that the protective benefit of estradiol lies in restoring both cellular signaling quality and the collagenous matrix structure.

Osteonecrosis has been reported in OVX models where ALN combines with corticosteroids ^45^ or infection,^33^, ^46^ though it also occurs with high ALN doses alone ^35^ or following the extraction of multiple adjacent molars.^13^ This study used lower doses, which did not induce osteonecrosis, supporting the idea that MRONJ risk escalates with dosage, duration, or additional factors. Our results aligned with previous reports that bisphosphonates alter initial healing of extraction sockets by suppressing bone turnover.^4^, ^5^ We observed similarly reduced bone formation and decoupled remodeling and provided the novel insight that this suppression is exacerbated in the absence of estrogen.

Estrogen deficiency after menopause can compromise oral health, increasing the risk of lesions and infections. HRT may reduce dry mouth and periodontitis.^47^, ^48^ Combining bisphosphonates and estrogen benefits women with severe osteoporosis due to their different mechanisms.^49^ Our research showed that HRT enhanced bone repair after tooth extraction in OVX rats treated with ALN. However, limited studies on this combination highlight the need for personalized approaches and further research.

From a translational perspective, these findings do not support prescribing hormone therapy by dental professionals but suggest a meaningful hypothesis: postmenopausal patients already on HRT who later require bisphosphonates may face a lower risk of alveolar repair complications than those receiving bisphosphonates alone. This distinction is important for preoperative risk stratification in implantology and dento‐periodontal surgery,^47^ reinforcing the need for close medical–dental collaboration to optimize outcomes.^1^

The study has limitations inherent to experimental design. Limitations include the single time‐point assessment and lack of micro‐CT or biomechanical analysis. However, the histomorphometry and immunohistochemical panel elucidated the cellular response, offering a meaningful translational perspective on how combined therapies may influence bone repair. Ultimately, this controlled model isolates the interaction between estrogen deficiency and bisphosphonates, generating the mechanistic hypothesis that background estrogen status may modulate the tissue response to antiresorptive therapy during healing.

## 5 CONCLUSION

In conclusion, chronic oral ALN treatment (5.0 and 10.0 mg/kg, twice weekly) was associated with reduced alveolar bone fill at day 28, an effect exacerbated by estrogen deficiency. HRT with E (0.8 mg/kg/day) partially mitigated these adverse effects of ALN on alveolar bone repair. The absence of frank osteonecrotic lesions indicates that this specific regimen did not induce necrosis; however, clear remodeling disturbances (impaired bone fill and failure to normalize molecular markers) were observed. These findings highlight the potential of estrogen replacement to modulate the tissue response to bisphosphonate treatment during bone repair.

## AUTHOR CONTRIBUTIONS

Pedro Henrique Chaves Isaias conceived and designed the study, acquired data, analyzed the data, drafted the article, and approved the final version. Paulo Goberlânio de Barros Silva conceived the study, analyzed the data, critically revised the manuscript, and approved the final version. Isabelly Vidal do Nascimento conceived the study, acquired data, and approved the final version. Raul Anderson Domingues Alves da Silva, Thayla Marla do Carmo Sousa, Ana Júlia Alves de Vasconcelos, and José Vitor Mota Lemos acquired data and approved the final version. Fabrício Bitu Sousa and Ana Paula Negreiros Nunes Alves conceived the study, acquired data, and approved the final version. Mário Rogério Lima Mota conceived and designed the study, analyzed the data, drafted the article, and approved the final version.

## CONFLICT OF INTEREST STATEMENT

The authors declare no conflicts of interest.

## FUNDING INFORMATION

The authors have nothing to report.

## Supporting information



Supporting information

## Data Availability

The data that support the findings of this study are available from the corresponding author upon reasonable request.
