# Supplementary material for: Estradiol replacement attenuates alendronate‐associated adverse effects on alveolar bone repair in ovariectomized rats
Source: J Periodontol. 2026 Feb 19;97(6):1271–84. doi: 10.1002/jper.70088 (PMC13350297; doi:10.1002/jper.70088)
Supplement: Supplementary file 1 — Supporting information [file JPER-97-1271-s001.docx]

**Estradiol replacement attenuates alendronate-associated adverse effects on alveolar bone repair in ovariectomized rats**

**Supplementary Figure S1**: Preparation process for histomorphometric analysis of bone marrow space area in the mandible and femur. The area of bone within the dental alveolus following the extraction of the left lower first molar, as well as the femoral head, was measured using Freehand selections followed by the Measure function (**A**). The region of interest was then isolated (Clear Outside; **B**), its colors inverted (Invert; **C**), and the RGB color channels adjusted (Color Threshold) to highlight the bone tissue while rendering the marrow area white (**D**). Subsequently, the image was converted to 8 bits (Image > Type > 8 bits; Image > Adjust > Threshold; **E**-**F**), ensuring that it was binarized (Process > Binary > Make Binary; **G**), and the black area was measured (Analyze > Analyze Particles; **H**).


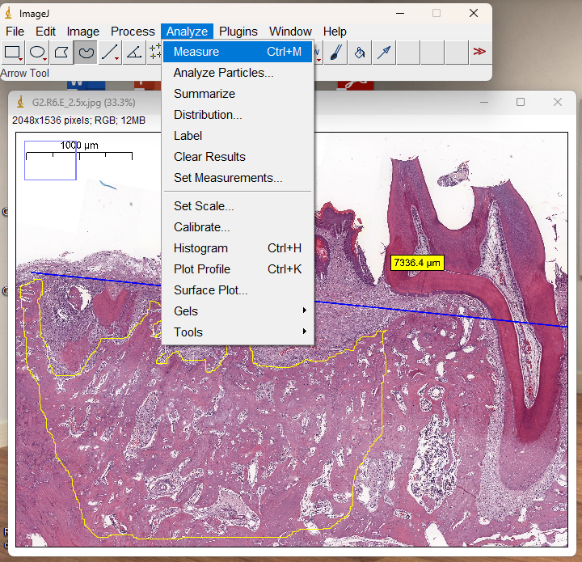

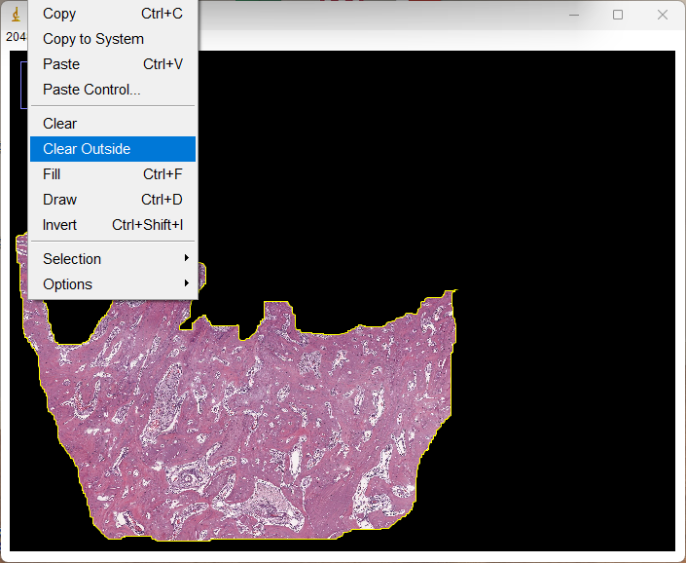


**A**

**B**


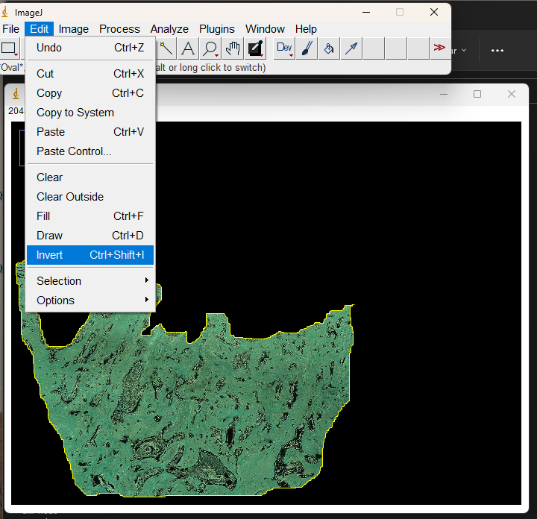

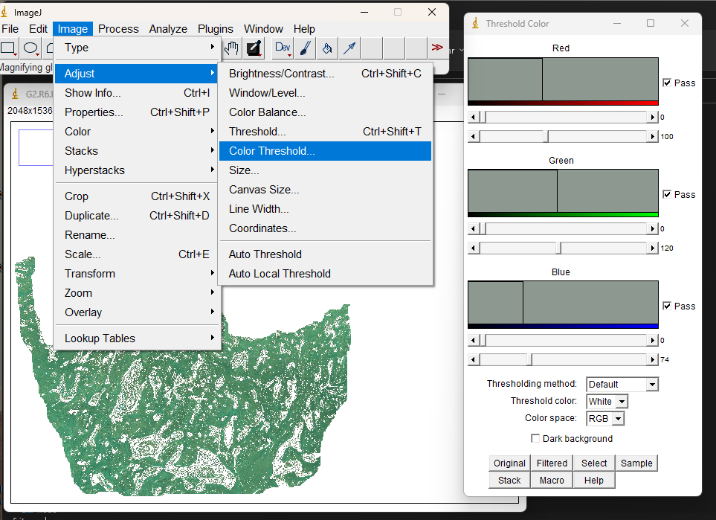


**D**

**C**

*Continuation of* ***Supplementary Figure S1****:*


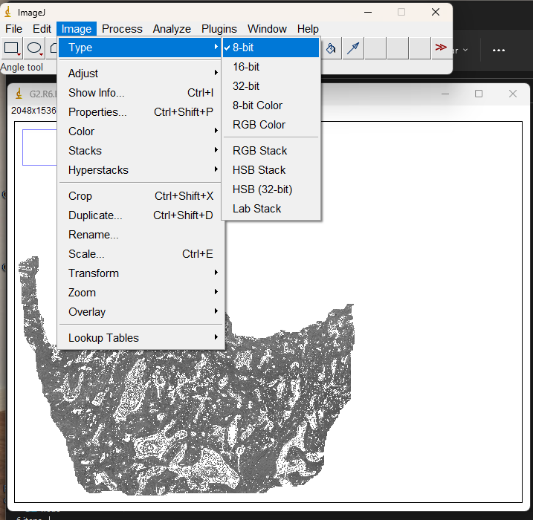

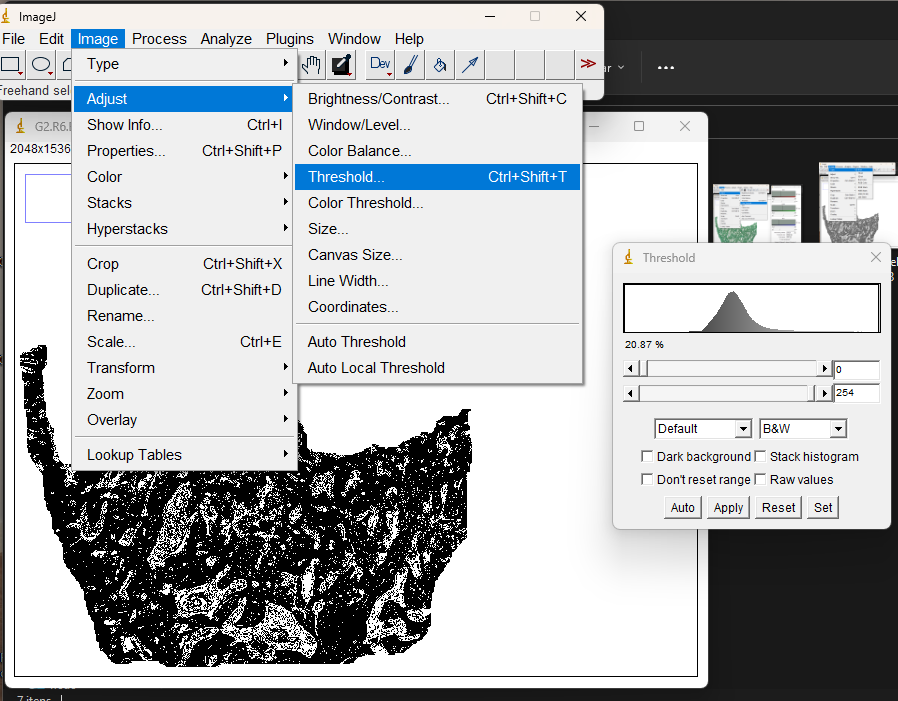

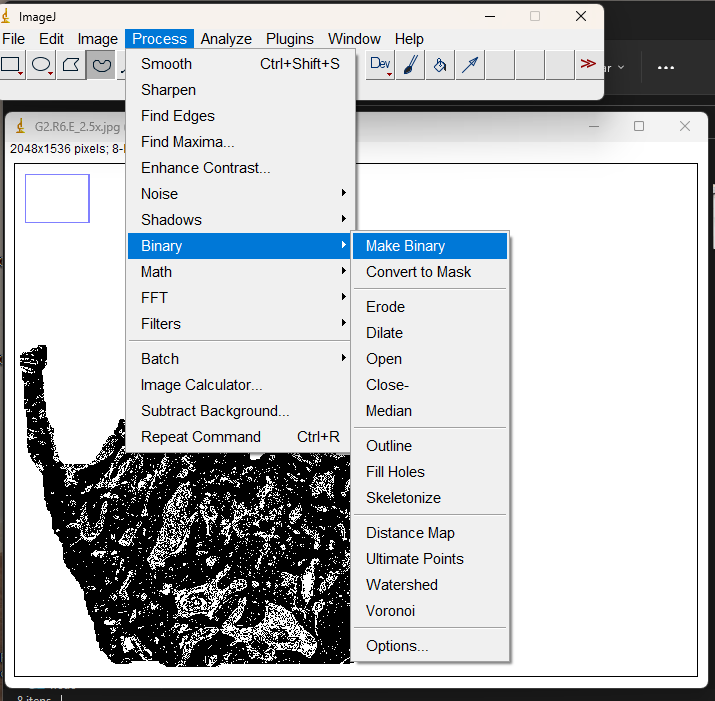

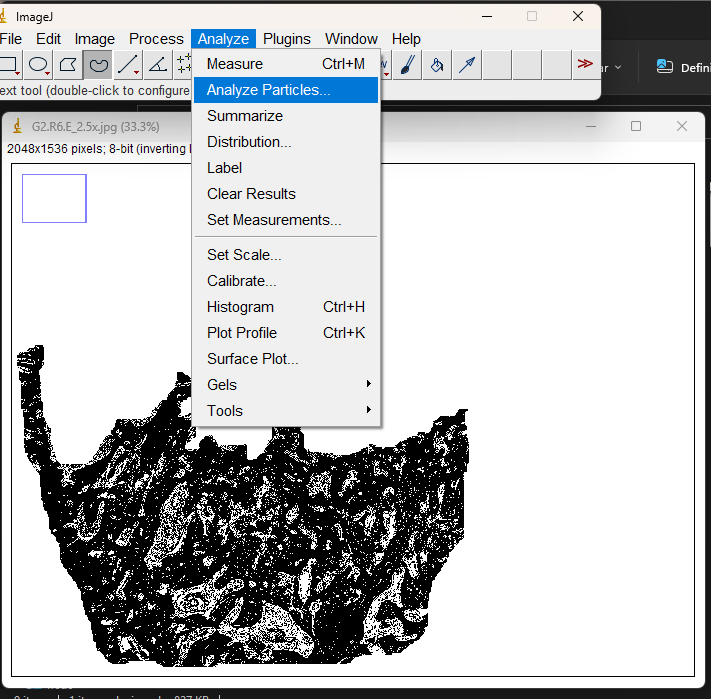


**H**

**G**

**F**

**E**

**Supplementary Figure S2**: Weekly progression of body mass in OVX animals, chronically treated with ALN with or without HRT using E and subjected to exodontia. The OVX was performed on day D_-56_, estrogen replacement was initiated on day D_-49_, ALN treatment commenced on day D_0_, exodontia was conducted on day D_42_, and the SHAM groups underwent simulated surgery without ovarian removal. OVX increased body mass in all animals subjected to this procedure starting from day D_-28_. *p<0.05 versus SHAM-SAL control group, analyzed using a 2-way ANOVA for repeated measures followed by Bonferroni post hoc test (mean ± SEM).


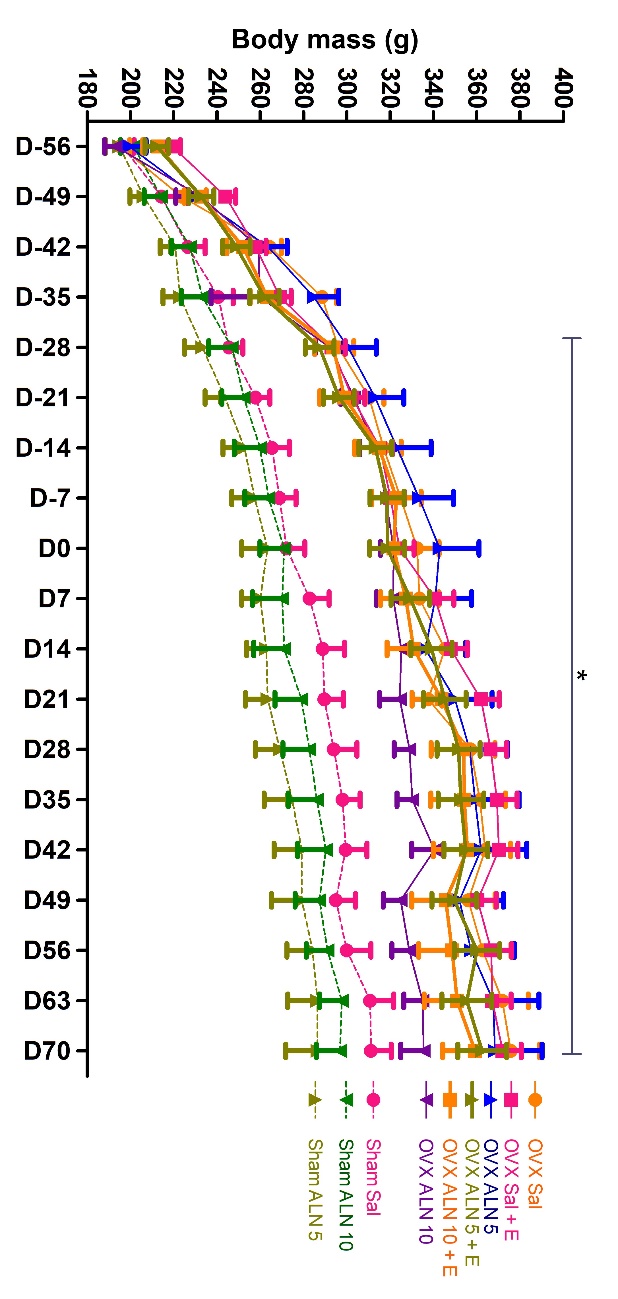


**Supplementary Figure S3**: Histological presentation of the femoral head in OVX animals, chronically treated with ALN, with or without HRT using E, and subjected to exodontia (hematoxylin and eosin, 50×).


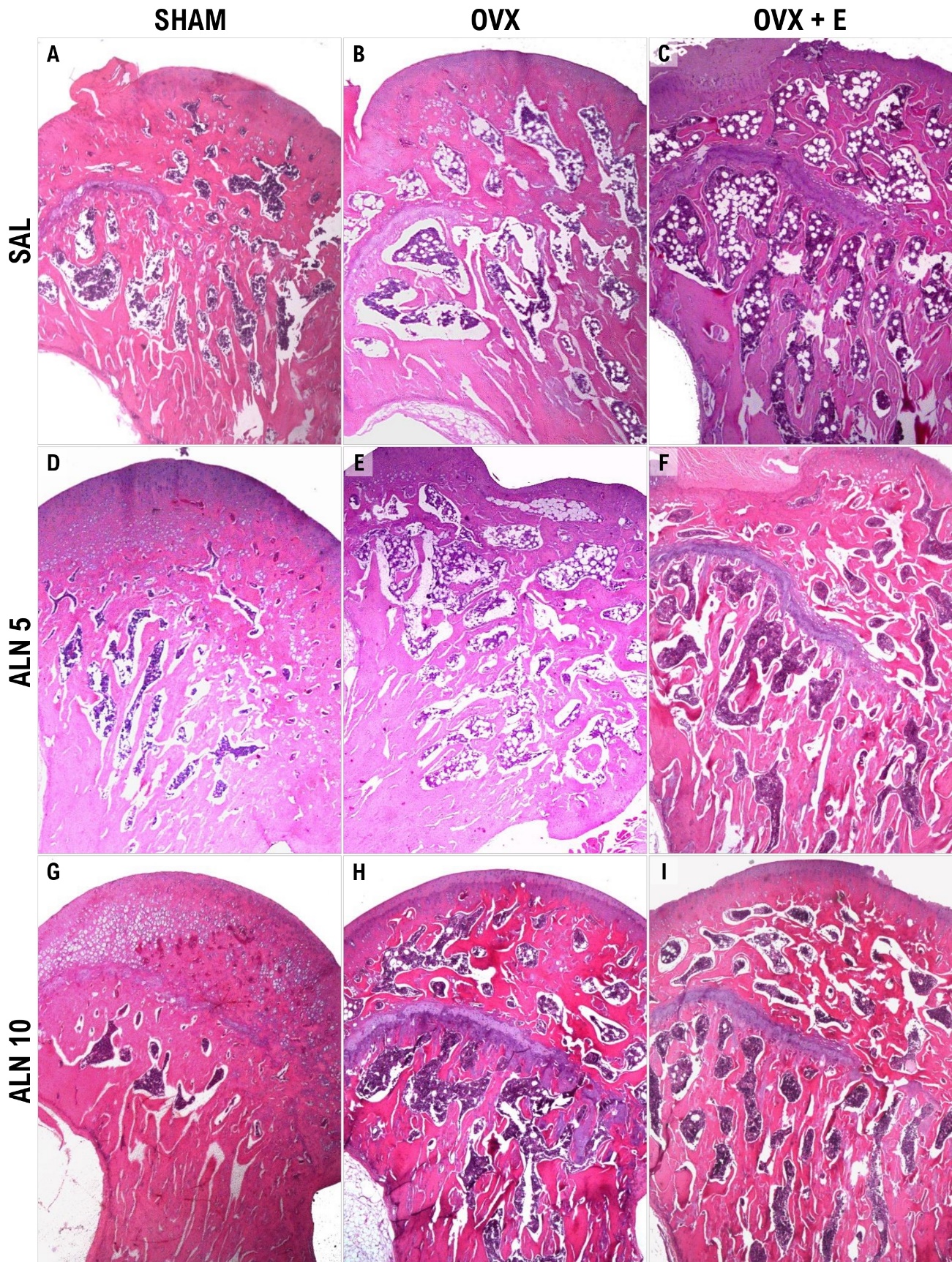


**Supplementary Table S1**: Wet uterine mass at the time of euthanasia, percentage of the medullary area in the femoral head, and total leukocyte count were evaluated in OVX animals, chronically treated with ALN, with or without HRT using E, and subjected to exodontia.

|  | **SHAM** | **OVX** | **OVX + E** | **p-Value I** | **p-Value OVX** | **p-Value ALN** |
| --- | --- | --- | --- | --- | --- | --- |
| Uterine mass (mg) |  | | | | | |
| **SAL** | 1.97±0.16 | 0.30±0.01*^†^ | 1.42±0.09* | 0.791 | ***<0.001*** | 0.524 |
| **ALN5** | 1.85±0.18 | 0.25±0.02*^†^ | 1.39±0.09* |  |  |  |
| **ALN10** | 1.71±0.04 | 0.30±0.02*^†^ | 1.40±0.08* |  |  |  |
| Femur medullary bone area (%) |  | | | | | |
| **SAL** | 14.42±0.97^●^ | 26.93±1.02* | 24.41±1.48* | ***0.001*** | ***<0.001*** | ***<0.001*** |
| **ALN5** | 15.35±1.02^●^ | 20.54±1.09*^●^ | 19.66±0.49*^●^ |  |  |  |
| **ALN10** | 14.39±1.25^●^ | 20.66±1.22*^●^ | 19.49±0.43*^●^ |  |  |  |
| Total leukocytes (n) |  | | | | | |
| **SAL** | 5083±541 | 5842±354 | 4571±472 | 0.136 | 0.860 | 0.187 |
| **ALN5** | 4679±418 | 5093±1030 | 6530±668 |  |  |  |
| **ALN10** | 4700±317 | 4971±566 | 6175±477 |  |  |  |
| ALN: sodium alendronate; E: estradiol valerate; OVX: ovariectomy; SHAM: sham-ovariectomy. Interaction (I) refers to OVX × ALN interaction. *p<0.05 versus SAL-SHAM control group; ^●^p<0.05 versus SAL-OVX group ; ^†^p<0.05 versus SAL-OVX+E group, 2-way ANOVA followed by Bonferroni post-test (mean±SEM). | | | | | | |
